# Detection of *KRAS* mutations in liquid biopsies from metastatic colorectal cancer patients using droplet digital PCR, Idylla, and next generation sequencing

**DOI:** 10.1371/journal.pone.0239819

**Published:** 2020-11-25

**Authors:** Matilda Holm, Emma Andersson, Emerik Osterlund, Ali Ovissi, Leena-Maija Soveri, Anna-Kaisa Anttonen, Soili Kytölä, Kristiina Aittomäki, Pia Osterlund, Ari Ristimäki

**Affiliations:** 1 Department of Pathology, Medicum, Faculty of Medicine, University of Helsinki and HUSLAB, HUS Diagnostic Center, Helsinki University Hospital, Helsinki, Finland; 2 Applied Tumor Genomics Research Program, Research Programs Unit, Faculty of Medicine, University of Helsinki, Helsinki, Finland; 3 Department of Surgery, Medicum, Faculty of Medicine, University of Helsinki and Helsinki University Hospital, Helsinki, Finland; 4 Translational Cancer Medicine Research Program, Research Programs Unit, Faculty of Medicine, University of Helsinki, Helsinki, Finland; 5 Department of Genetics, HUSLAB, HUS Diagnostic Center, Helsinki University Hospital and University of Helsinki, Helsinki, Finland; 6 Department of Immunology, Genetics and Pathology, Uppsala University, Uppsala, Sweden; 7 Department of Radiology, HUS Diagnostic Center, Helsinki University Hospital, and University of Helsinki, Helsinki, Finland; 8 Department of Oncology, Clinicum, Helsinki University Hospital and University of Helsinki, Helsinki, Finland; 9 Hyvinkää Hospital and Hyvinkää Homecare, Hyvinkää, Finland; 10 Department of Oncology, Tampere University Hospital, Tampere, Finland; 11 Faculty of Medicine and Life Sciences, Tampere University, Tampere, Finland; Peter MacCallum Cancer Institute, AUSTRALIA

## Abstract

Circulating tumor DNA (ctDNA) is released from cancer cells and oncogenic mutations in ctDNA can be measured from plasma samples. Droplet digital PCR (ddPCR) is a sensitive and specific method for the detection of mutations in ctDNA. We analyzed serial plasma samples (n = 80) from ten metastatic colorectal cancer (mCRC) patients with a known *KRAS* mutation in their primary tumor. The patients were undergoing oncological treatment with bevacizumab in combination with alternating capecitabine and oxaliplatin or irinotecan. Baseline ddPCR *KRAS* mutation allele frequency (MAF) values ranged from 0% to 63%. The first radiologic response evaluation criteria in solid tumors (RECIST) evaluation was performed 45–63 days after the initiation of treatment, and by this time three patients had an undetectable level of *KRAS* mutation, one had a MAF value of 0.5%, and one had a MAF value of 3% that had been reduced by 95% from the baseline value. In three of these patients the RECIST assessment was stable disease and in two partial response. In seven patients, ddPCR MAF values increased before radiological disease progression or death, while one patient remained disease-free with an undetectable *KRAS* mutation level. Next, we analyzed all available plasma samples with the Idylla ctKRAS system (n = 60), and found that the overall degree of agreement between ddPCR and Idylla was almost perfect (kappa value = 0.860). We used next-generation sequencing (NGS) to detect treatment-induced mutations in the last serial plasma sample of each patient, but were unable to find any new mutations when compared to the primary tumor. This study shows that ddPCR and Idylla are equally efficient for the detection of *KRAS* mutations in the liquid biopsies from mCRC patients and that ctDNA may indicate the disappearance of treatment responsive *KRAS* positive mCRC clones and serve as an early sign of disease progression.

## Introduction

Cell-free DNA (cfDNA) is released into the circulation by both physiological and pathological mechanisms. In cancer patients, a fraction of blood-borne cfDNA is tumor-derived and called circulating tumor DNA (ctDNA) [1]. The measurement of mutations in ctDNA from liquid biopsies, which provide a minimally invasive way to collect samples such as plasma, requires sensitive techniques. Droplet digital PCR (ddPCR) is a sensitive, specific, and accurate method that enables the detection and quantitation of targeted DNA mutations in a variety of clinical samples [2]. Next generation sequencing (NGS), however, has its benefits in detecting different types of mutations, including novel ones, but does not usually reach the level of sensitivity achieved using ddPCR [1, 3]. The Idylla system (Biocartis, Mechelen, Belgium) is a fully automated, real-time PCR-based molecular diagnostics system that can quickly and without pre-analytical DNA extraction identify oncogenic mutations, such as in the *KRAS* gene, in tissue and plasma samples [4].

The analysis of ctDNA provides the opportunity to identify the genetic changes during the carcinogenic cascade. In a metastatic setting, periodically collected plasma samples and analysis of ctDNA mutations therein can be used to monitor tumor burden, therapy response, and relapse [4]. However, there is currently a lack of sufficient evidence and guidelines to support the use of ctDNA in clinical practice for gastrointestinal cancers [5]. Furthermore, widespread utilization of ctDNA as a predictive marker for personalized therapy in oncology is still very limited, although the FDA has recently approved a liquid biopsy test to detect *EGFR* mutations in non-small cell lung cancer patients and *PIK3CA* mutations in breast cancer as predictive markers [6, 7].

In metastasized colorectal cancer (mCRC), the application of liquid biopsies with the most potential is to monitor treatment response and to detect relapse at an earlier point in time than current clinical, laboratory or imaging modalities are able to. To this end, it is important to pay attention to sensitivity and specificity and to perform cross-platform comparison studies [8, 9]. The aim of this study was to compare multiple methods for measuring *KRAS* mutations in periodically collected liquid biopsies. Here we have collected serial plasma samples (n = 80) from ten mCRC patients and analyzed them using ddPCR, Idylla, and NGS platform.

## Materials and methods

### Study design and patients

This study used samples from patients included in the AXOAXI trial (NCT01531595 and EudraCT 2011-003137-33), which was a multicenter, open-label, single-arm phase II study of first- and second-line bevacizumab treatment in combination with alternating capecitabine and oxaliplatin (B-CAPOX) or irinotecan (B-CAPIRI) [10]. Treatment response evaluation according to RECIST 1.1 was performed every 9 weeks by thoracic, abdominal and pelvic computed tomography (CT)-scan. Plasma samples were collected at baseline (before initiation of the treatment), after the first cycle (at three weeks), during treatment response evaluation (every 9 weeks) during first-line and at progression during later line. The primary endpoints of the AXOAXI study were 12-month progression free survival rate and resectability of metastases, while secondary endpoints included biomarker evaluation. Eligibility criteria were metastatic adenocarcinoma of colorectal origin, no previous chemotherapy for mCRC, age over 18, Eastern Cooperative Oncology Group performance status 0–2 and adequate bone marrow, kidney and liver function. The Ethical Review Board at Helsinki University Hospital approved the protocol and written informed consent was obtained from all patients.

The ten patients included in this study are a subset of the 77 patients recruited to the study. Inclusion criteria were a previously known *KRAS* mutation in the primary CRC resection specimen and adequate plasma collection, processing, and storage. We were able to retrieve 5–12 samples from each patient and the baseline samples were available for six of the patients. All included patients were from the Helsinki University Hospital and treated between April 2015 and March 2017. The patient characteristics are shown in Table 1. Data points are shown in S1 Table.

**Table 1:** Clinical data of the patients in this study.

Patient number	Age	Gender	Primary location	Surgery of primary	Prior adjuvant chemo- or radiotherapy	Presentation of metastases	Liver metastases	Lung metastases	Peritoneal metastases	KRAS mutation	Metastasectomies
1	57	Female	Left	Yes	No	Synchronous	Yes	Yes		G12D	
2	64	Male	Left	Yes	No	Synchronous	Yes	Yes	Yes	G13R	
3	38	Male	Right	No	No	Synchronous			Yes	G12D	
4	68	Male	Left	No	No	Synchronous	Yes			G12V	
5	62	Female	Left	Yes	No	Synchronous		Yes		G12C	
6	76	Male	Right	Yes	No	Synchronous	Yes			G12D	Liver x1
7	45	Male	Rectum	No	No	Synchronous	Yes			G12D	
8	47	Male	Left	Yes	No	Synchronous	Yes			G12D	Liver x3
9	66	Male	Right	Yes	Yes	Metachronous			Yes	G13D	
10	66	Female	Right	No	No	Synchronous	Yes			G12C	

### Sample preparation

Blood samples were collected in EDTA-tubes and centrifuged within 30 min to extract plasma. The extracted plasma was immediately stored at -20°C and moved to -80°C within two weeks. For ddPCR and next-generation sequencing (NGS) analysis, DNA was extracted from 2 ml of plasma with the QiaSymphony SP, following the protocol for the QiaSymphony DSP Circulating DNA Kit (Qiagen, Hilden, Germany).

### Droplet digital PCR

Targeted wild-type and mutation probes for the *KRAS*-mutations 12 and 13 were designed and prevalidated by Bio-Rad (www.biorad.com) (S2 Table) and 2 μl of the extracted DNA was used for each triplicate reaction. The QX200 Droplet Generator partitioned the samples (20 μl into 20,000 droplets) for PCR amplification. Following amplification using a thermal cycler, droplets from each sample were analyzed individually on the QX200 Droplet Reader, where PCR-positive and PCR-negative droplets were counted to provide absolute quantification of the target DNA in digital form. The results were analyzed with the QuantaSoft Analysis Pro Software (v.1.0, Bio-Rad, Hercules, CA, USA).

### Idylla

Plasma (1 ml) was loaded into the Idylla^TM^ ctKRAS cartridge together with 50 μl of proteinase K-enzyme. The Idylla^TM^ ctKRAS Mutation Test (Biocartis, Mechelen, Belgium) covers 21 different *KRAS* mutations in exons 2, 3 and 4 [11].

### Next-generation sequencing

Next-generation sequencing (NGS) using an in-house cancer panel of seven target genes (*PIK3CA*, *EGFR*, *KIT*, *KRAS*, *MET*, *NRAS*, and *PDGFRA*) and exons 11–15 of *BRAF* was performed on samples taken from the primary tumor of all patients [12]. The KRAS primers for the codons 12 and 13 mutations were designed on the Ion AmpliSeq Designer (Thermo Fisher Scientific, Waltham, MA, USA). Positions of used primers were NM_033360.3(KRAS):g.25398162_25398185 and g.25398387-25398415. A more comprehensive NGS analysis was subsequently performed on the last serial plasma sample taken from each patient using the Ion AmpliSeq Hotspot Panel v2, which surveys the hotspot regions of 50 oncogenes and tumor suppressor genes (Thermo Fisher Scientific). If mutations that had not been detected in the initial analysis of the primary tumor were found in the plasma sample, the primary tumor was also analyzed using the Ion AmpliSeq Hotspot Panel v2. Libraries were pooled and loaded onto the Ion PI^TM^ Chip using the Ion Chef^TM^ instrument and sequenced with the Ion Proton^TM^ System (Thermo Fisher Scientific). The limit of detection of the NGS platform is 1–2%.

### Data analysis

DdPCR values were associated with patients’ treatment response according to RECIST 1.1 (blinded review by AO), CEA values, and plotted against time in graphs. Graphs were drawn using GraphPad Prism (GraphPad Software version 8.0.2, La Jolla, California, USA). The degree of agreement between ddPCR and Idylla was quantified by kappa (GraphPad QuickCalcs; https://www.graphpad.com/quickcalcs/kappa1/). The associations between ddPCR MAF versus CEA and CA19-9 were studied in the ten patients, producing a total of 75 observations. Because of repeated measurements for each patient, analysis of variance (ANOVA) with the generalized estimating equations (GEE) analyses and an unstructured working correlation models were used. The distributions of CEA and CA19-9 were skewed to the right and were logarithmically transformed (ln) before analysis. The CA19-9 values below the detection limit were replaced by detection limit/2. About one half of ddPCR MAF values were zero. Thus, the associations were not studied including ddPCR MAF as a continuous variable. Instead, we divided the values of independent variable ddPCR MAF into three categories (0, 0.2–9.9, ≥10%) and used ln(CEA) and ln(CA19-9) as dependent variables. The results for each category given by generalized estimation equation (GEE) analysis are described as geometric means (95% CI). Analyses were performed using IBM SPSS Statistics for Windows (version 26.0, Armonk, NY, USA, IBM Corp.). A p-value of less than 0.05 was considered statistically significant. For calculating correlation in other datasets, the Spearman coefficient was used (GraphPad Prism).

## Results

### Patient characteristics

Ten mCRC adenocarcinoma patients were included in this study (Table 1). The median age of the patients (three female and seven male) was 63 years (range 38–76 years). The primary tumor was right-sided in four and left-sided in six. The primary tumor was operated in six patients. The metastases were synchronous in nine and metachronous in one (peritoneal spread). The most frequent metastatic site was the liver (n = 7), followed by the lung (n = 3) and the peritoneum (n = 3). Two patients had metastases in multiple organs and two patients underwent resection of their liver metastases. All of the patients had a previously diagnosed *KRAS* mutation in codon 12 or 13 in the primary tumor (Table 1). Patient #6 was alive at data cut-off 13^th^ May 2020 while the median overall survival was 21.6 months (range 12.7–51.4) for the rest of the nine patients.

### DdPCR analysis of *KRAS* mutations in plasma samples of mCRC patients

Eighty serially collected plasma samples were drawn from the ten mCRC patients (5–12 samples per patient) in this study. CfDNA was extracted from all plasma samples (n = 80) and analyzed by ddPCR targeting the *KRAS* mutations that had earlier been diagnosed in the primary tumor.

**Fig 1:**
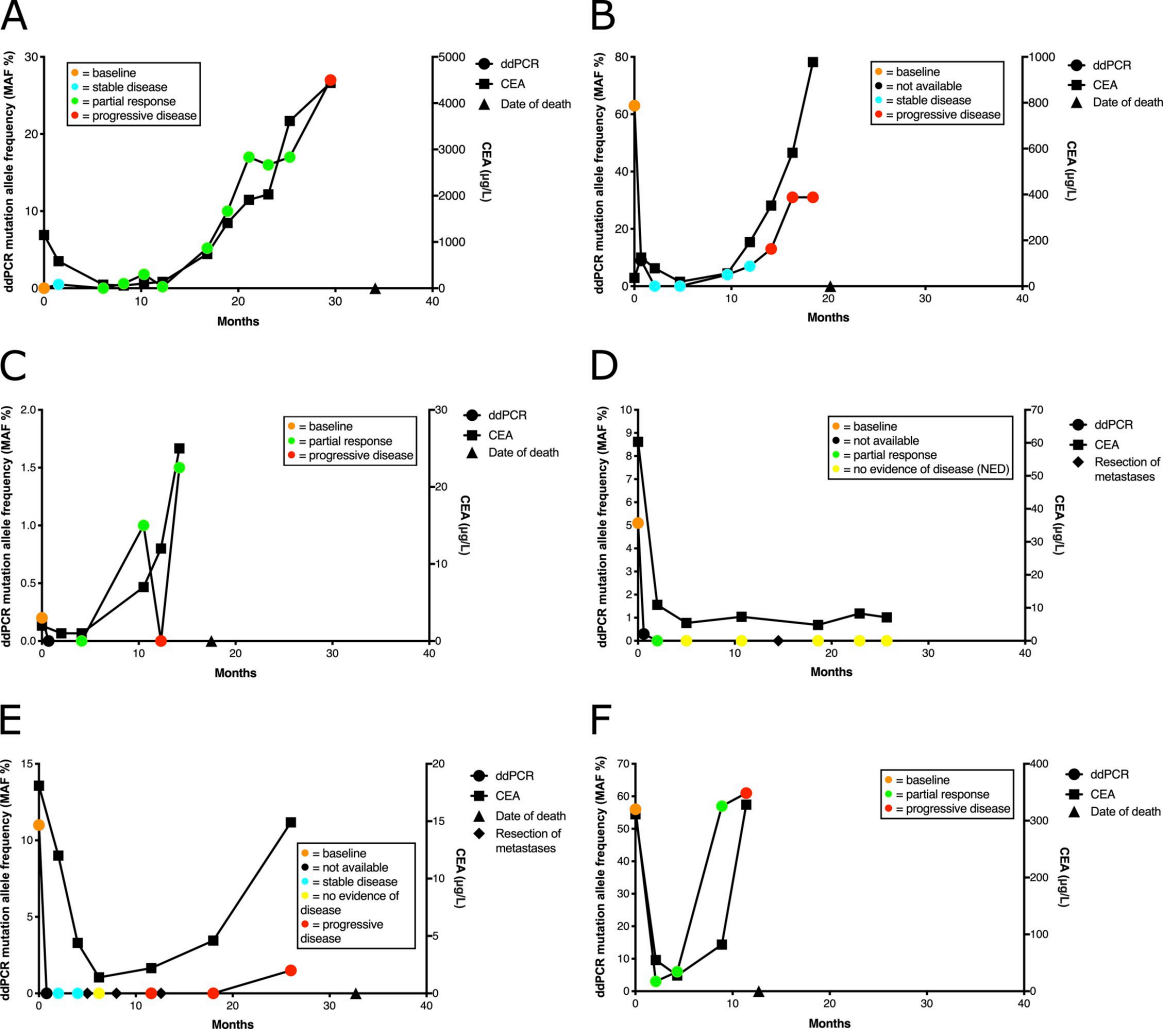
DdPCR *KRAS* mutation allele frequency (MAF) of serial plasma samples, RECIST evaluation, and CEA values of six patients. DdPCR MAF values (%) are shown on the left y-axis and CEA measurements on the right y-axis for patients #1 (A), #4 (B), #5 (C), #6 (D), #8 (E), and #10 (F). Baseline is depicted by orange circles (0 day time point) and RECIST evaluation of stable disease by blue, partial response by green, progressive disease by red, and no evidence of disease by yellow. CEA values are depicted with solid squares. The triangle on the x-axis indicates the time of death. The rhombuses on the x-axes Fig 1D and 1E) indicate the resection of liver metastases. Patient #6 (Fig 1D) is the only long-term survivor in the cohort.

DdPCR *KRAS* mutation allele frequency (MAF) values of the full plasma series from six patients (#1, #4, #5, #6, #8, and #10) are shown in Fig 1, along with CEA values and RECIST objective response rate (ORR). Baseline MAF values were low in patients #1 and #5 (Fig 1A and 1C; 0% and 0.2%, respectively), intermediate for patients #6 and #8 (Fig 1D and 1E; 5.1% and 11%) and high for patients #4 and #10 (Fig 1B and 1F; 63% and 56%). The MAF values for the only long-time survivor (patient #6) fell to zero by the time of the first RECIST evaluation (partial response) at 58 day time point after the initiation of treatment (Fig 1D). In this patient, CEA levels also fell sharply, but remained at a measurable level during the follow-up (7.1 μg/ml at the 771 day time point). The survival times from baseline for the other patients (#1, #4, #5, #8 and #10) in Fig 1 were 34.1, 20.2, 17.5, 32.7, and 12.7 months, respectively. There was no correlation between baseline MAF values and overall survival (p = 0.45, Spearman correlation). However, the only patient (#10) whose MAF value did not decrease to an undetectable level had the shortest overall survival, while the patient (#1) whose baseline MAF value was zero had the longest survival. The data of the remaining four patients, from whom baseline plasma samples were not available, are shown in S1 Fig. Patients #3 and #9 (S1B and S1D Fig) only had peritoneal metastases with ddPCR values of zero throughout the follow-up, although the disease was fatal.

**Fig 2:**
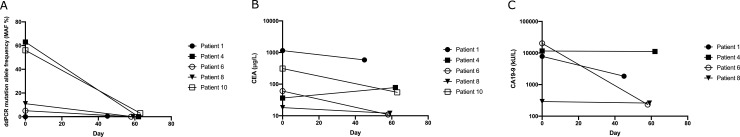
DdPCR *KRAS* mutation allele frequency (MAF; A), CEA (B), and CA19-9 (C) values of mCRC patients from baseline to the first sample collected with RECIST evaluation of five patients. Baseline values are shown at the zero day time point. Filled symbols indicate a RECIST evaluation of stable disease, while open symbols indicate a RECIST evaluation of partial response. Patient #10 is not shown in Fig 2C, as the CA19-9 levels for this patient were below the detection limit (<2 kU/L) at both baseline and at all follow-up time points.

Next, we evaluated ddPCR *KRAS* MAF, CEA, and CA19-9 values at the first RECIST ORR evaluation time point, which was 45–63 days after initiation of the first trial treatment. Fig 2A shows MAF values from 0% to 63%, with the median value being 11%. A clear reduction in ddPCR MAF values was observed at the time of the first RECIST evaluation, as MAF values were zero (patients #4, #6, and #8) or very low (0.5% for patient #1). In the remaining patient (#10), values had decreased by 94.6% (from 56% to 3.0%). For patient #5, no ctDNA was available for ddPCR analysis at the appropriate time point (61 d), and this patient was not included in Fig 2. Two patients (#6 and #10) showed partial response, while the rest of the patients (#1, #4, and #8) had a stable disease. The corresponding reductions in the sum of target lesions at first response evaluation were -2.5%, -34%, -13%, -18% and -44% (for patients #4, #6, #8, #1, #10). For CEA the corresponding figures were +117%, -82%, -33%, -49% and -82% and for CA19-9–3.6%, -68%, -11%, -76% and not measurable.

CEA and CA19-9 values are shown for the baseline and for the first RECIST evaluation follow-up time point in Fig 2B and 2C (CA19-9 levels for patient #10 were below the detection limit, which was <2 kU/L, at both baseline and at all follow-up time points). A clear reduction in CEA and CA19-9 values (81.7–98.9%) was seen for the two patients (#6 and #10) who had a partial response and whose ddPCR MAF values were zero (patient #6) or reduced by 94.6% (patient #10). In patients #1 and #8, who had stable disease at this time point, the reduction of CEA and CA19-9 values was more modest (10.7–76.5%) and in one patient (#4), whose MAF values became zero, showed an increase in CEA values without any clear reduction in CA19-9 values. There was a significant association between ddPCR MAF values and CEA (p < 0.001). Geometric means of CEA were 14 (95% CI 6.9–29), 78 (34–180) and 133 (57–309) in the ddPCR MAF categories 0, 0.2–9.9 and ≥10.0%, respectively. This correlation was evident in most patients at the time of disease progression, although in patient #7 (S1C Fig), the increase in ddPCR MAF values was clearer than that of CEA (the increase of MAF was 26.7 fold and that of CEA 3.3 fold when the lowest follow-up value was compared to the last sample of the series). In addition, for the peritoneal carcinomatosis patients #3 and #9, whose MAF values were zero, CEA values were at a clearly detectable level (S1B and S1D Fig). The association between ddPCR MAF and CA19-9 had the same direction as CEA, but was not significant. The geometric means of CA19-9 were 103 (23–448), 163 (34–769) and 223 (61–814), respectively (p = 0.19).

### Concordance in *KRAS* mutation status between ddPCR and Idylla

**Fig 3:**
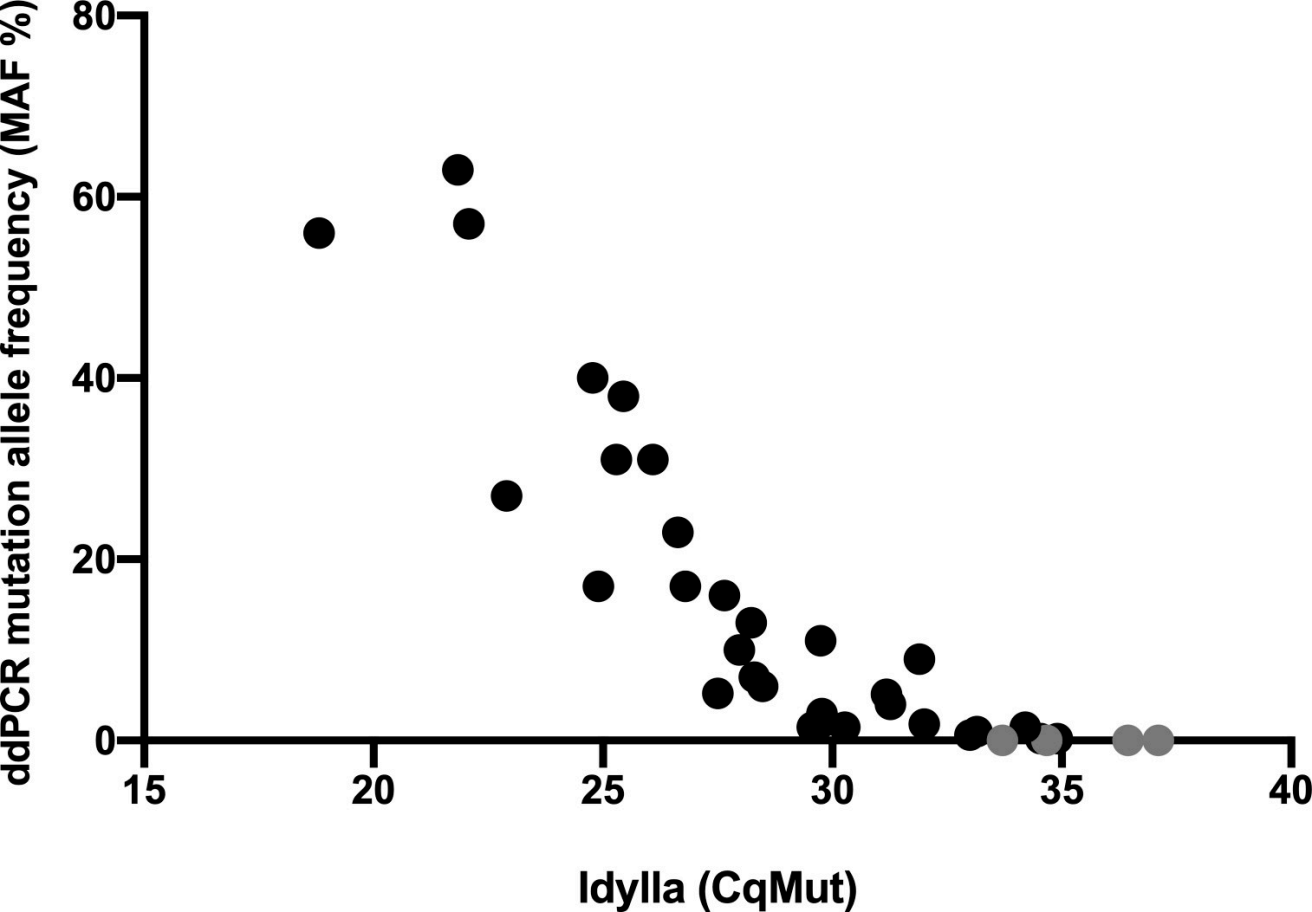
Concordance in *KRAS* mutation status between ddPCR and Idylla. Mutations in *KRAS* codons 12 or 13 were analyzed from the plasma samples (n = 60) of nine mCRC patients. CfDNA was extracted from 2 ml of plasma for ddPCR analysis, while proteinase K-treated plasma (1 ml) was analyzed using Idylla. Four Idylla-positive samples gave a zero value when analyzed by ddPCR (marked in gray). Two samples gave an invalid result when analyzed by Idylla and are not depicted in the graph. The Spearman correlation coefficient between ddPCR and Idylla CqMut values was -0.9461 (95% CI -0.9732 –-0.8930, p < 0.0001), showing a strong inverse correlation between ddPCR MAF values and Idylla CqMut values.

DdPCR analysis detected *KRAS* mutations in 40/80 (50%) of the plasma samples, of which six had MAF values below 1%, 11 from 1% to 5%, and 23 over 5% (range 5.1–63%). Next, we analyzed the plasma samples with the Idylla ctKRAS Mutation Assay. One patient (#2), from which 12 plasma samples had been obtained, had a *KRAS* mutation (Gly13Arg) that is not included in the Idylla cartridges. Of the remaining 68 plasma samples, we were able to retrieve 60 for Idylla analysis. Of these, 58 (97%) were successfully analyzed, while two gave an invalid result. *KRAS* mutations were detected in 35/58 samples (60%). Four samples were positive with Idylla that had MAF values of zero with ddPCR. However, these samples showed high CqMut values (33.7–37.1) in the Idylla analysis, indicating a low mutation frequency (Fig 3). The degree of agreement (positive versus negative, kappa analysis, Table 2) for the two methods was 0.860 (95% CI 0.799–0.991), indicating an almost perfect concordance between the two methods. The Spearman correlation coefficient between ddPCR *KRAS* MAF values and Idylla ctKRAS CqMut values was calculated to be -0.9461 (95% CI -0.9732 –-0.8930, p < 0.0001), showing a strong agreement between the two methods. For the two peritoneal carcinomatosis patients (#3 and #9) that delivered a zero value when analyzed by ddPCR, Idylla showed a negative result in all but one sample, which had a CqMut value of 36.44.

**Table 2:** Contingency table of *KRAS* mutation status as analyzed by ddPCR and Idylla.

		Idylla	
		Positive	Negative
**ddPCR**	Positive	31	0
	Negative (MAF 0%)	4	23

### NGS analysis of *KRAS* mutations in plasma samples of mCRC patients

NGS was performed on the last serial plasma sample taken from each patient using the AmpliSeq Hotspot Panel v2 to analyze if any new mutations had appeared during the course of treatment. *KRAS* mutations were detected in 7 of the 10 mCRC patients using the hotspot panel. The three patients who did not have detectable *KRAS* mutations were patients #3 and #9, who displayed peritoneal carcinomatosis, and patient #6, who remained disease-free during the follow-up (these samples were negative also when analyzed using Idylla or ddPCR). Subsequently, the hotspot NGS panel was also performed on primary tumor samples in those cases where new mutations compared to the primary analysis were detected in the plasma. However, no new detectable mutations had arisen during treatment (Table 3).

**Table 3:** NGS results showing the mutations detected by the clinical or the hotspot panel in samples from the primary tumor and the last serial plasma sample.

	Mutations detected		
Patient	Clinical panel	Hotspot panel	Hotspot panel
	(primary tumor)	(plasma)	(primary tumor)
1	KRAS Gly12Asp	KRAS Gly12Asp	KRAS Gly12Asp
		PIK3CA Pro421A	PIK3CA Pro421A
2	KRAS Gly13Arg	KRAS Gly13Arg	KRAS Gly13Arg
		TP53 Cys238Tyr	TP53 Cys238Tyr
3	KRAS Gly12Asp	No mutations detected	Not performed
4	KRAS Gly12Val	KRAS Gly12Val	KRAS Gly12Val
		TP53 Arg196Ter	TP53 Arg196Ter
5	KRAS Gly12Cys	KRAS Gly12Cys	Not performed
6	KRAS Gly12Asp	No mutations detected	Not performed
7	KRAS Gly12Asp	KRAS Gly12Asp	KRAS Gly12Asp
		TP53 Leu111Arg	TP53 Leu111Arg
8	KRAS Gly12Asp	KRAS Gly12Asp	Not performed
9	KRAS Gly13Asp	No mutations detected	Not performed
10	KRAS Gly12Cys	KRAS Gly12Cys	KRAS Gly12Cys
		TP53 His193Arg	TP53 His193Arg

## Discussion

In this study, we analyzed *KRAS* mutations in serial plasma samples from mCRC patients using ddPCR, Idylla, and NGS. Four of 35 Idylla positive samples were at undetectable range (MAF zero) when analyzed by ddPCR and the Idylla CqMut values for these four cases were high, indicating a low mutation load. All ddPCR positive samples were positive when analyzed using Idylla and thus, there was a strong agreement between the two methods. The analytical sensitivity of Idylla in detecting *KRAS* mutations has been reported to be in the range of 0.1–1% and that of digital PCR platforms as low as 0.001–0.01% [2, 11, 13]. In agreement, Vessies *et al*. recently reported that ddPCR is more sensitive when compared with Idylla for detecting *KRAS* mutations in plasma samples from mCRC patients [14]. However, it is currently unknown which level of sensitivity is required in a clinical setting for the follow-up of mCRC patients using liquid biopsies to determine treatment response or relapse. In our experience, Idylla is at least as sensitive and specific as ddPCR in detecting *KRAS* mutations in plasma samples and its advantage is extended to a fast workflow without the need for preanalytical DNA extraction. However, ddPCR has a clear advantage over Idylla as quantitative MAF values can be obtained, whereas Idylla delivers only a readout of positive or negative as a CE-IVD certified readout. Also, two samples that were successfully analyzed using ddPCR gave an invalid result using Idylla, and thus the rate of non-analyzable plasma samples was 3.3%. In addition, mutations that are not included in the Idylla cartridge are readily detectable using ddPCR, which also offers analytical options beyond mutation allele quantitation, such as the analysis of copy number variations, DNA methylation, and gene rearrangements [2]. Finally, when four different analytical platforms were compared, Idylla was the least expensive in a low throughput analytical setting, while ddPCR was the least expensive in a higher sample throughput setup [14].

A strength of this study is the well-characterized patient cohort, for which sample collection was well-documented. Treatment response was analyzed in a blinded manner and follow-up data over a long period of time was available. The quantitative property of ddPCR was further utilized to understand treatment response and effect on survival. Limitations of the study included a low number of patients and the selective nature of the study setup. In our material, there was no correlation between baseline MAF values and overall survival. However, there was a clear decrease in MAF values in each of the four patients who had a measurable level of the mutation in the baseline sample and the patient whose MAF values did not decrease to an undetectable level during the serial sample collection had the shortest overall survival. This may indicate that the unresponsive *KRAS* mutated clones harbor a detrimental role. We observed a significant association between ddPCR *KRAS* MAF values and CEA measurements when the associations were studies using GEE analysis (p < 0.001). However, when the first response to treatment evaluation was performed, in one patient ddPCR values become zero with a clear increase of CEA and without a significant decrease in CA19-9 measurements, and in several patients the reduction in ddPCR values was more pronounced when compared to these classic serum tumor markers. These differences may at least partially depend on the short half-life of ctDNA in the blood when compared to the protein tumor markers [15]. Although *KRAS* MAF values decreased dramatically, only two patients were shown to have a radiological response (PR), while three of the patients had stable disease according to the radiological RECIST assessment. It is therefore possible that *KRAS* MAF values mirror a different biological phenomenon than the RECIST assessment. Interestingly, the disappearance of *RAS* mutations in plasma has been reported in some patients with primarily *RAS* mutant cancers, which raises the question of whether liquid biopsy testing might expand the population of anti-EGFR-eligible patients by including those with primary *RAS*-mutant mCRC [16, 17].

The main disadvantage of ddPCR and Idylla is their limited capacity to analyze multiple different kinds of genomic alterations simultaneously. NGS therefore has the advantage that no prior knowledge of the molecular alteration is needed. Additionally, NGS can detect the presence of both somatic and germline mutations, copy number alterations, and other chromosomal alterations, such as transversions, inversions, and translocations [18]. However, NGS is a more time-consuming and expensive method than ddPCR and Idylla. NGS is most often used for analysis of the primary tumor mutation spectrum, and Idylla liquid biopsy results show an overall agreement of 73–82% when compared with standard tissue-based NGS analysis, which was increased to 96–100% for patients with liver metastases [19, 20]. In our series, all mCRC patients had been diagnosed with *KRAS* mutations in their primary tumor using a limited clinical NGS cancer panel. We analyzed the last plasma sample of each patient using the Ion AmpliSeq Hotspot Panel v2, which surveys the hotspot regions of 50 oncogenes and tumor suppressor genes, and found a *PIK3CA* mutation in one patient and *TP53* mutations in four patients. However, we did not find any novel mutations compared to the primary tumors.

A study by Cao *et al*. used a 605-gene panel to study mutations in both plasma ctDNA and CRC tissues at baseline and after first-line treatment (chemotherapy plus bevacizumab and/or cetuximab). This study discovered new mutations in *ATM* and *NF1* after treatment and a new mutation in *PDGFRB* during disease progression [21]. A study by Yamauchi *et al*. analyzed plasma samples and pre-treatment tumor samples from 21 mCRC patients undergoing treatment with bevacizumab and oxaliplatin- or irinotecan-based chemotherapy. The authors performed NGS using a panel of 90 oncogenes and detected new mutations, one in *CREBBP* and one in *FBXW7*, in the ctDNA from two patients [22]. Although these two studies report the discovery of new treatment-induced mutations at a low frequency, a study by Osumi *et al*. did not discover any new mutations after treatment with bevacizumab and chemotherapy, although the authors suggested that changes in ctDNA levels may be useful to predict the outcomes of mCRC patients [23]. Patients treated with anti-EGFR therapy have been shown to acquire resistance through the emergence of KRAS mutations [24]. Treatment with bevacizumab does not seem to induce mutations in the same way, although increased levels of VEGFR1 can lead to bevacizumab resistance [25]. New mutations may have been discovered in our study if a wider NGS panel had been used. However, as all seven patients with liver and/or lung metastases in our series showed an increase of *KRAS* MAF values upon disease progression (RECIST PD or death), this indicates that a *KRAS* mutation alone is quite an effective marker of progressive disease. More importantly, while CEA values remained at measurable level, the only long-term survivor in our series showed non-measurable levels of KRAF MAF in all follow-up samples in the no evidence of disease period. Of note is also that the two patients with only peritoneal metastases did not have a detectable *KRAS* mutation in the ddPCR analysis. This is in accordance with data published by Vivancos *et al*. [11], who showed that patients with only peritoneal metastases have the lowest frequency of *KRAS* mutations in liquid biopsy samples when compared with those that have liver and/or lung metastases. Especially in these patients, CEA seems to be a better measure of tumor load when compared to detection of *KRAS* mutations in ctDNA.

Techniques such as Idylla and ddPCR have recently come into the spotlight as ways to efficiently measure ctDNA mutations from liquid biopsies. This study showed that Idylla is at least as sensitive as ddPCR in detecting previously known *KRAS* mutations in the plasma cfDNA of mCRC patients. However, ddPCR offers a clear advantage over Idylla due to its ability to deliver MAF values and wider range of target mutations. It will be important to further investigate whether metastases of *RAS*-mutated primary tumors can be converted to *RAS* wild-type, which could possibly lead to their sensitization to EGFR-targeted treatments. Importantly, an increase in *KRAS* mutation load in ctDNA is a signal of disease progression in most mCRC patients.

## Supporting information

S1 Fig DdPCR *KRAS* mutation allele frequency (MAF) of serial plasma samples, RECIST evaluation, and CEA values of four patients. DdPCR MAF values (%) are shown on the left y-axis and CEA measurements on the right y-axis for patients #2 (A), #3 (B), #7 (C), and #9 (D). RECIST evaluation of stable disease is depicted by blue, partial response by green, progressive disease by red, and no evidence of disease by yellow. CEA values are depicted with solid squares. The triangle on the x-axis indicates the time of death (in Fig A, the OS for patient #2 was 51.4 months).
(TIFF)

S1 Table The Idylla, ddPCR, CEA, and CA19-9 values analyzed in this study. (XLSX)

S2 Table KRAS primers and probe sequences used for ddPCR analysis (proprietary to Bio-Rad). (XLSX)
