# Supplementary figures and images for: Detection of KRAS mutations in liquid biopsies from metastatic colorectal cancer patients using droplet digital PCR, Idylla, and next generation sequencing
Source: PLoS One. 2020 Nov 25;15(11):e0239819. doi: 10.1371/journal.pone.0239819 (PMC7688175; doi:10.1371/journal.pone.0239819)

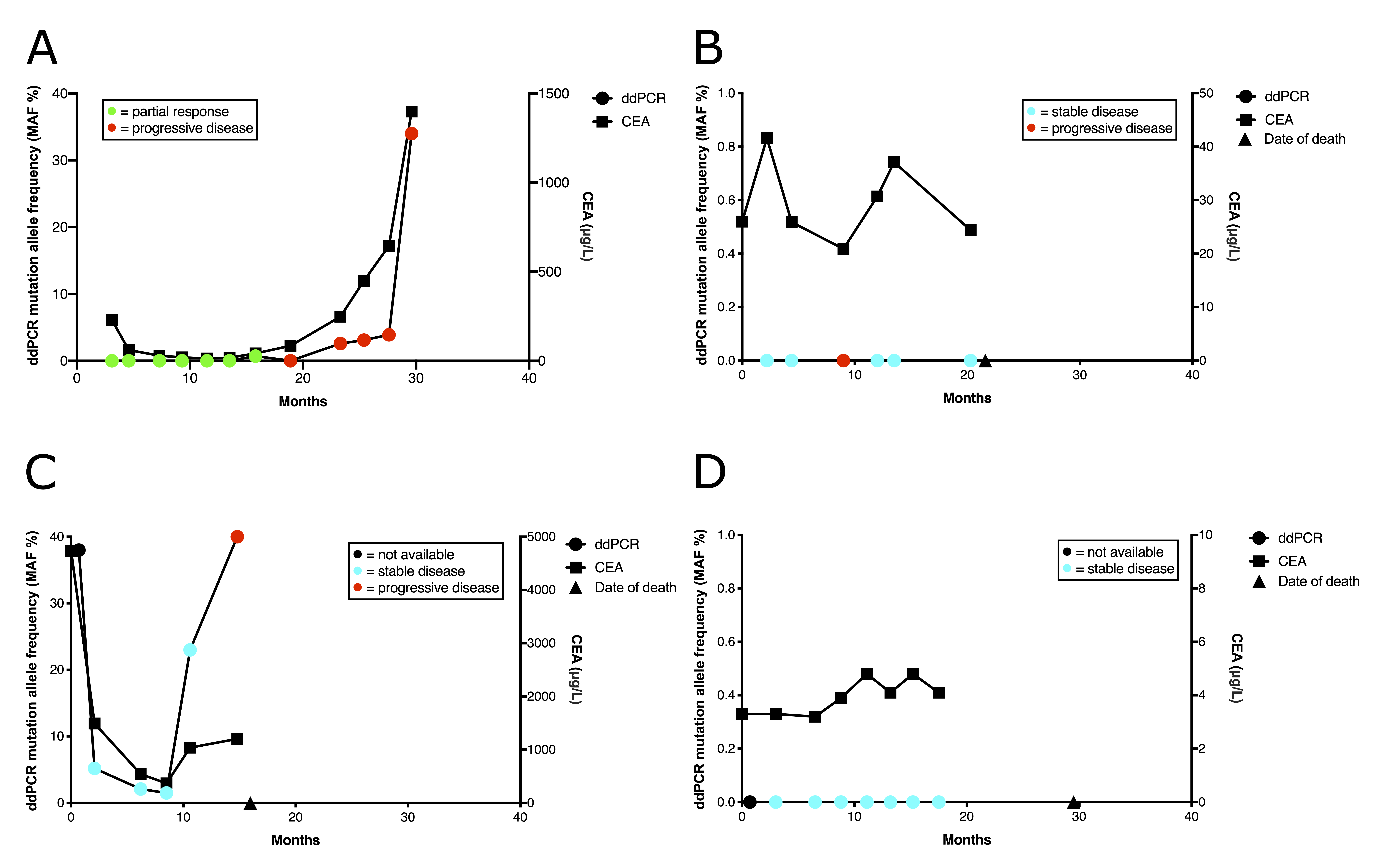

Supplement: S1 Fig — DdPCR MAF values (%) are shown on the left y-axis and CEA measurements on the right y-axis for patients #2 (A), #3 (B), #7 (C), and #9 (D). RECIST evaluation of stable disease is depicted by blue, partial response by green, progressive disease by red, and no evidence of disease by yellow. CEA values are depicted with solid squares. The triangle on the x-axis indicates the time of death (in Fig A, the OS for patient #2 was 51.4 months). (TIFF) [file pone.0239819.s001.tiff]
